# Supplementary material for: Prognostic analyses of genes associated with anoikis in breast cancer
Source: PeerJ. 2023 Oct 11;11:e15475. doi: 10.7717/peerj.15475 (PMC10576492; doi:10.7717/peerj.15475)
Supplement: Table S2 [file peerj-11-15475-s002.docx]

| Gene Symbol | | | | | |
| --- | --- | --- | --- | --- | --- |
| BIRC3 | SERPINA1 | CD24 | FGFR1 | IVL | KRT15 |
| L1CAM | TP63 | MIA | NDRG1 | NOS2 | PTPN3 |

### **Table 1. List of gene symbol of anoikis-related genes of LASSO model.**
