# Supplementary figures and images for: Prognostic analyses of genes associated with anoikis in breast cancer
Source: PeerJ. 2023 Oct 11;11:e15475. doi: 10.7717/peerj.15475 (PMC10576492; doi:10.7717/peerj.15475)

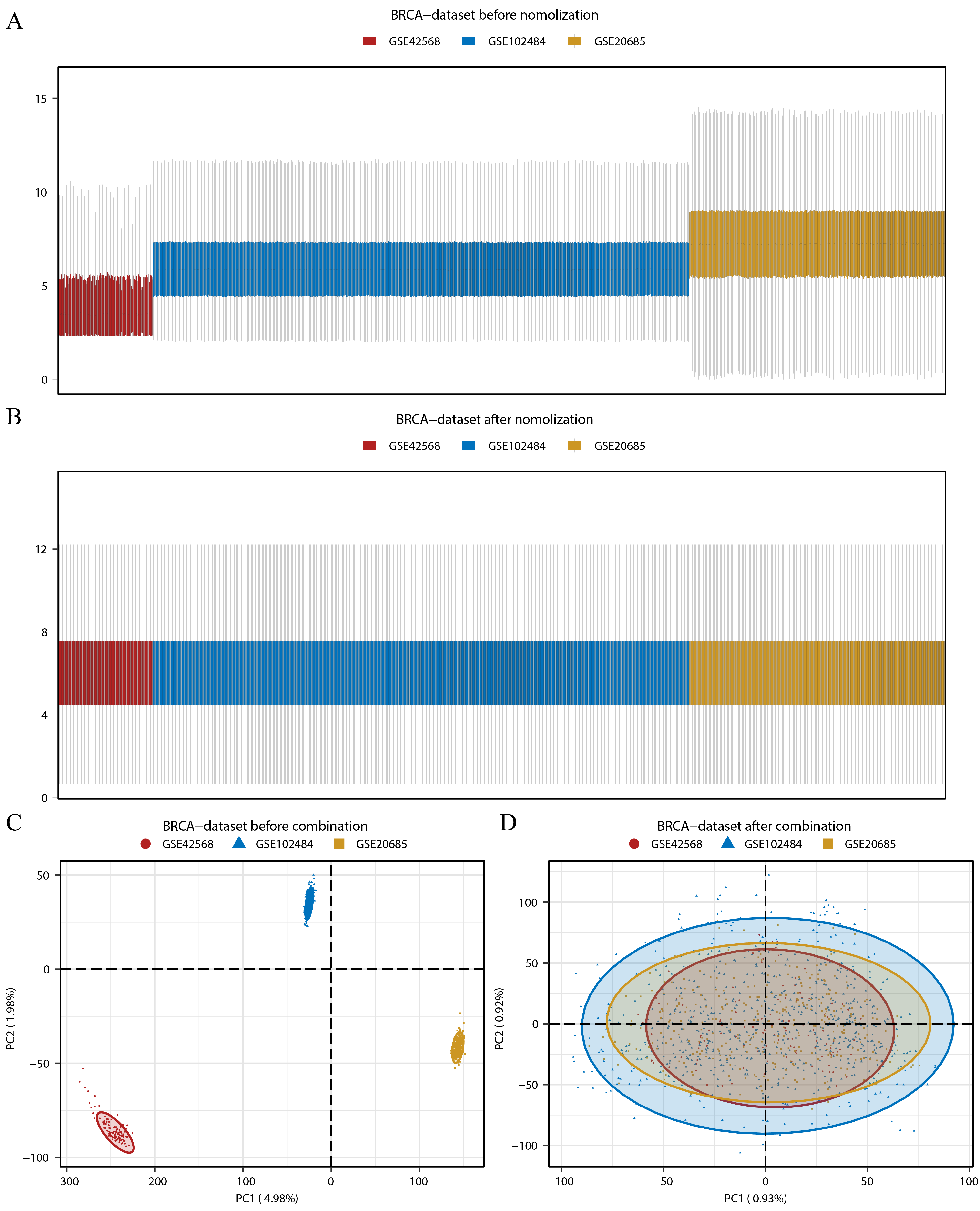

Supplement: Figure S1 — (A–B) Box plots displayed the BRCA dataset before (A) and after the elimination of batch effects (B). (C–D) PCA plots displayed the BRCA dataset before (C) and after the elimination of batch effects (D). BRCA, breast invasive carcinoma; PCA, principal component analysis. [file peerj-11-15475-s003.png]

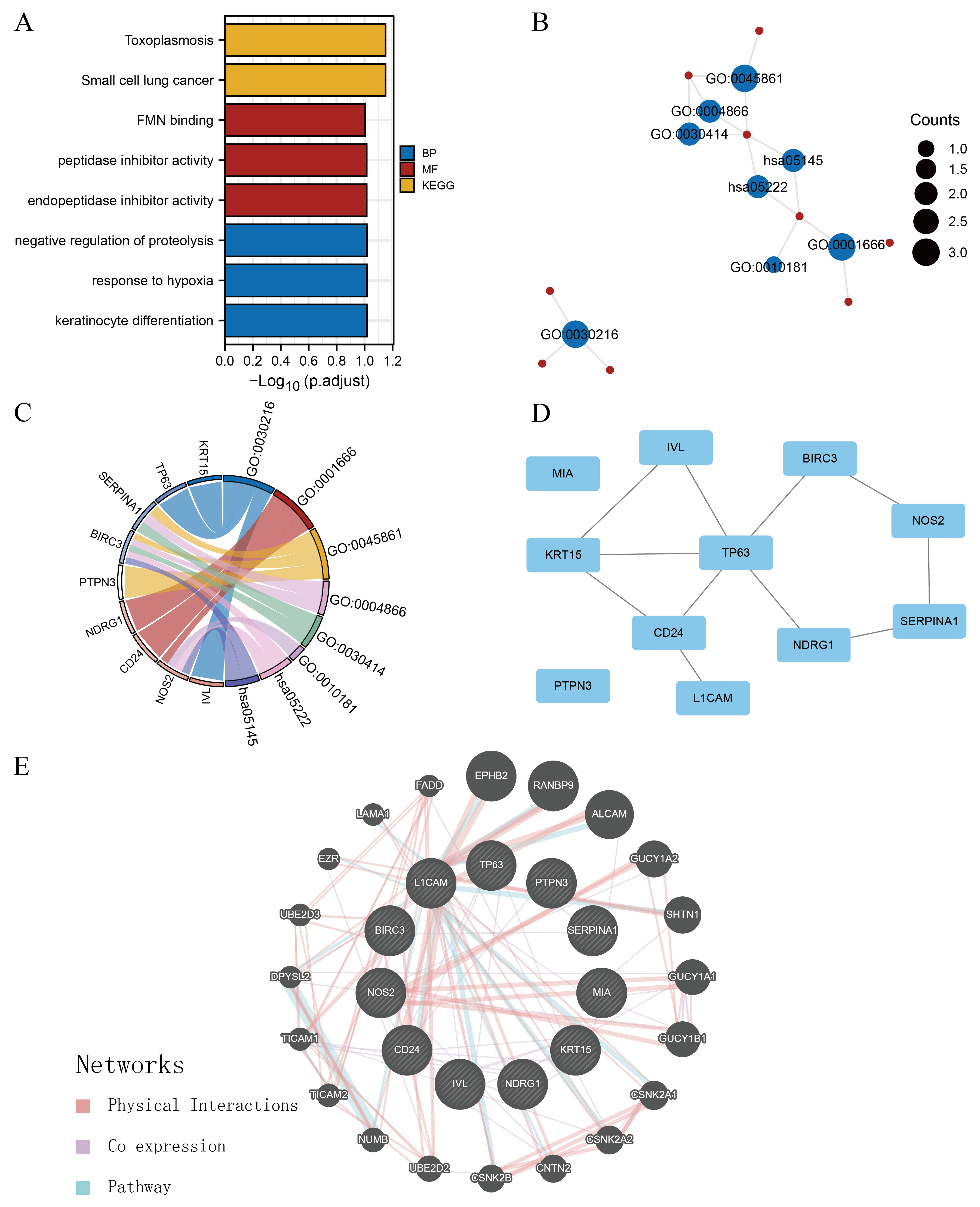

Supplement: Figure S2 — (A–B) The histogram and network diagrams indicate GO and KEGG analyses of 11 ARGs. Red and blue dots represent genes and pathways, respectively. (C) Chordal graph of GO and KEGG analyses of ARGs combined with logFC. (D) PPI network of ARGs constructed using Cytoscape based on the STRING database. (E) PPI network of ARGs and related genes based on GeneMANIA. PPI, protein-protein interaction; GO, Gene Ontology; KEGG, Kyoto Encyclopaedia of Genes and Genomes; ARGs, anoikis-related genes; STRING, Search Tool for the Retrieval of Interacting Genes/Proteins. [file peerj-11-15475-s004.png]

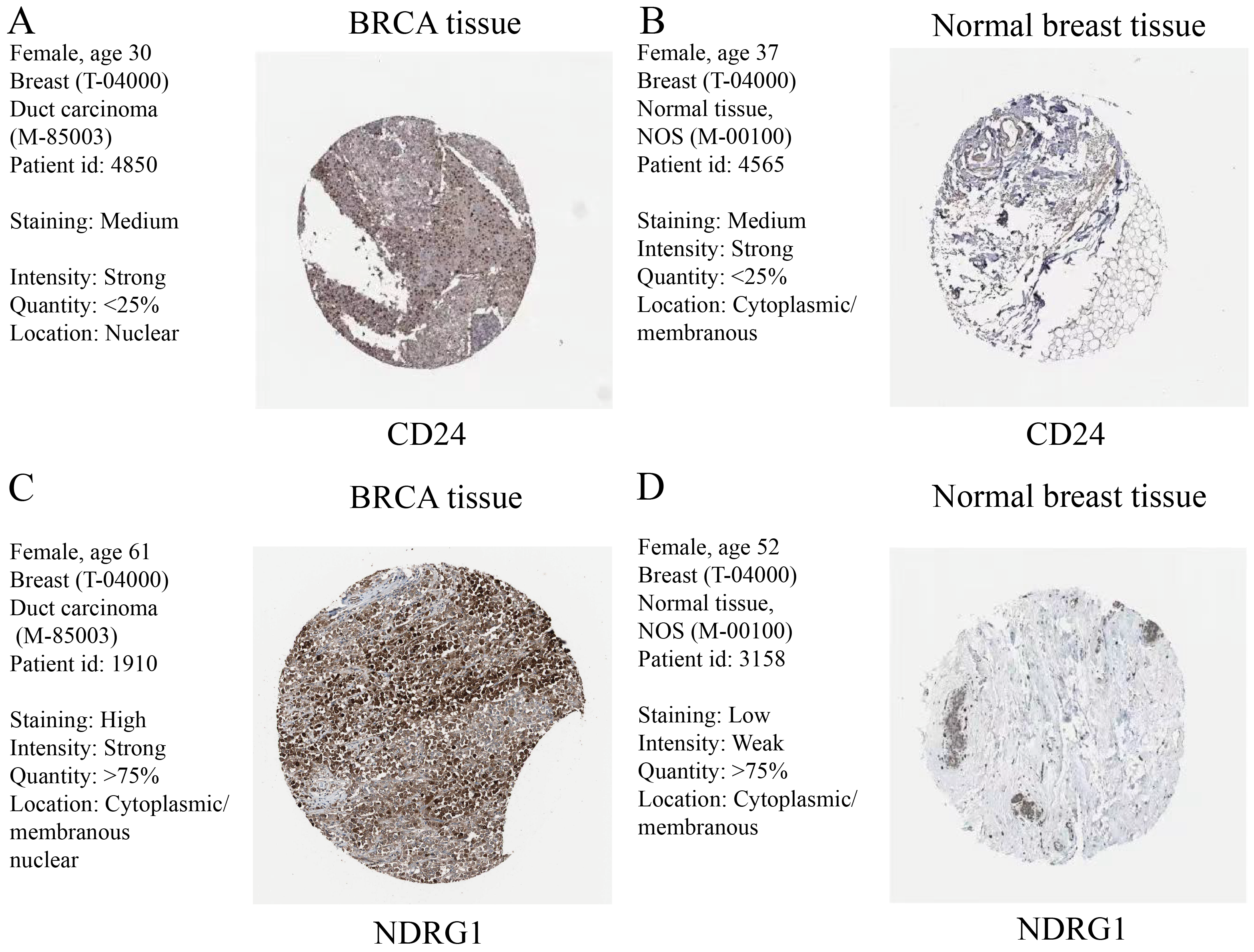

Supplement: Supplemental Information 5 — (A–B) CD24 protein levels in breast cancer tissue (A) and normal breast tissue (B). (C–D) NDRG1 protein levels in breast cancer tissue (C) and normal breast tissue (D). IHC, immunohistochemical; CD24, cluster of differentiation 24; NDRG1, N-Myc downstream regulated 1; BRCA, breast invasive carcinoma; HPA, human protein atlas. [file peerj-11-15475-s005.png]

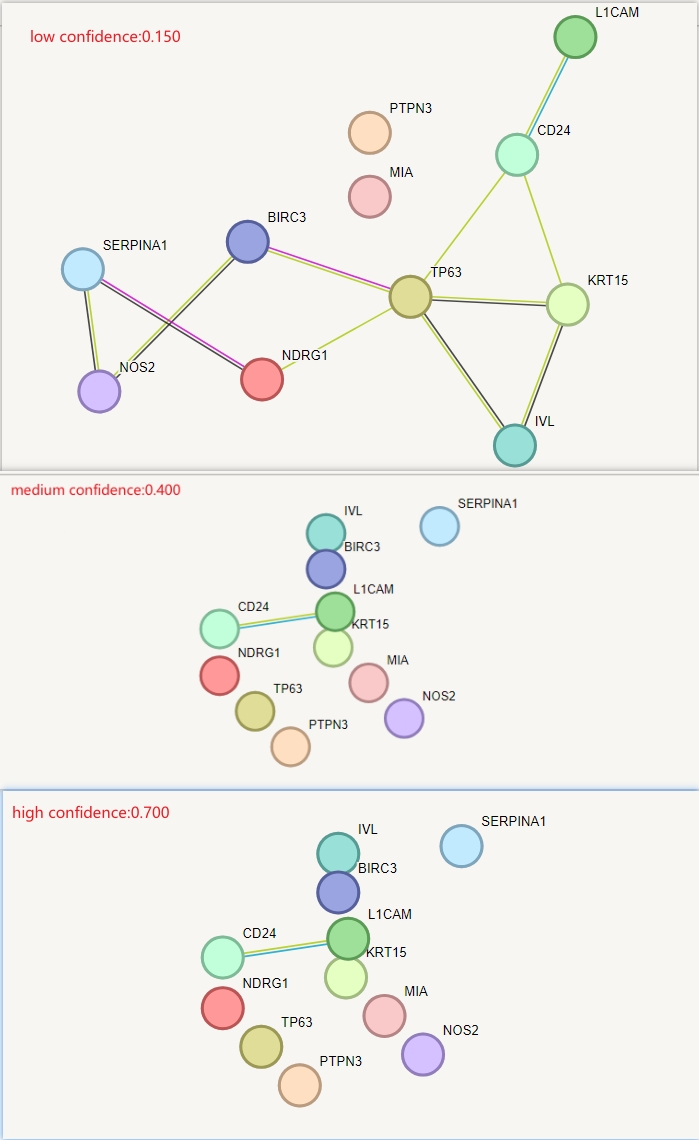

Supplement: Figure S1A [file peerj-11-15475-s006.jpeg]

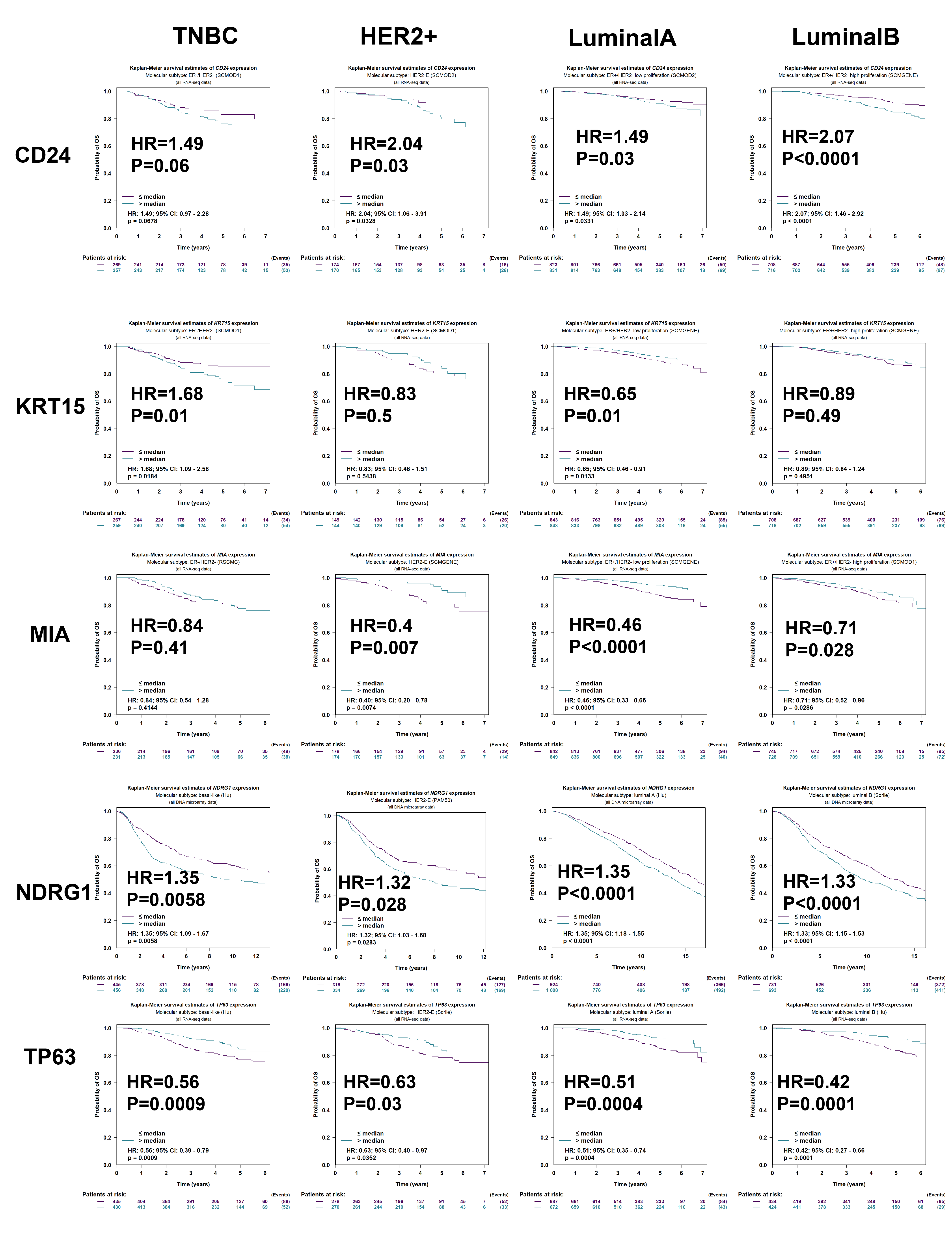

Supplement: Figure S2A [file peerj-11-15475-s007.png]
